# Present Evidence of Determinants to Predict the Efficacy of Renal Denervation

**DOI:** 10.1155/2022/5694127

**Published:** 2022-08-12

**Authors:** Hao Zhou, Yanping Xu, Weijie Chen, Liang Wang, Huaan Du, Hang Liu, Zhiyu Ling, Yuehui Yin

**Affiliations:** Department of Cardiology, the Second Affiliated Hospital of Chongqing Medical University, Chongqing, China

## Abstract

Sympathetic overactivation is one of the main contributors to development and progress of hypertension. Renal denervation (RDN) has been evidenced by series of clinical trials for its efficacy and safety to treat overactivated sympathetic nervous system induced diseases. However, the results were inconsistent and not all patients benefited from RDN. Appropriate patient selection and intraoperative factors to improve the efficacy of RDN need to be solved urgently. Over the decade, research studies on the correlations between indicators and the antihypertensive effects have been conducted and made a fairly well progress. Herein, we comprehensively reviewed the research studies on how to make RDN more predictable or improve the efficacy of RDN and summarized these potential indicators or devices which might be applied in clinical settings.

## 1. Introduction

Sympathetic overactivity is a major drive of initiation and development of many cardiovascular diseases [1]. However, therapeutic options to reduce sympathetic nervous activity are still very limited. From macroscope [2], laparoscope [3], to intervention [4, 5], renal denervation (RDN) has emerged as a novel nonpharmacological approach to effectively improve cardiovascular diseases by attenuation of sympathetic activities, such as heart failure [6], cardiac hypertrophy [7], arrythmias [8, 9], and, especially, resistant hypertension [4, 5, 10–14]. Although the negative results of blood pressure-lowering effects between the denervated and sham group by radiofrequency [15] and ultrasound [16] dampened the enthusiasm of researchers, growing evidence has recertified the efficacy of catheter-based renal denervation subsequently using either radiofrequency [10–13], ultrasound [14], or chemical ablations [17], which brought it back to the center of hypertension-therapeutic arena.

As reported in the previous clinical trials above, although the responders received a significant reduction of blood pressure after RDN and the efficacy exists persistently [18, 19] (up to 9-year follow-up [20]), there were about 25–30% nonresponders manifesting unchanged or elevated blood pressure postoperatively, which runs counter to our therapeutic purpose and burdens the patients. Therefore, indicators before/during RDN procedure become particularly important and indispensable to screen susceptible subjects, especially those with high sympathetic tone, leading to more predictable results and avoiding harm to patients. Herein, a comprehensive review was made to retrospect existing evidence on predicting the effectiveness of RDN.

## 2. Patient Selection

### 2.1. Renal Artery Anatomy

Sufficient diameter and length of renal arteries have been considered to be technically eligible for catheter-based renal denervation. Previous clinical studies [4, 5, 13, 15] have excluded patients with multiple renal arteries, while subsequent trials [10–12] reserved these patients. For those patients, Verloop et al. performed RDN on patients with accessory renal arteries [21] and concluded that the blood pressure-lowering effect of RDN in patients with multiple renal arteries (both main and accessory arteries were eligible for ablation) was similar to those with solitary renal arteries, but patients whose accessory arteries were less than 20 mm length or 4 mm diameter (unable to perform RDN) showed a trend to a less pronounced effect of RDN. As is reported by Sato et al. [22], there were considerable quantity of nerve bundles around accessory renal arteries in man with closer distance to adventitia and the number of renal nerves was dependent on the diameters of accessory artery/percentage of blood supply, although the number was less than that of dominant renal artery. This work could provide a better understanding to improve the operation of RDN.

Also, Uei Pua et al. [23] reported two cases with insufficient renal artery length (defined as less than 20 mm length), both of who manifested a 20–30 mmHg reduction of office blood pressure after RDN without addition of antihypertensive medications. Although the sample size was small, these studies might somehow relax the exclusion criteria.

### 2.2. Race

Since HTN-3 reported the differential effects of renal denervation on Americans versus African-Americans, several studies have examined the importance of race on the response to renal denervation. In the HTN-3 trial, the change of office SBP at 6-month follow-up in the RDN group, compared to the sham group, was 2.25 mmHg (95% confidence interval, CI, −7.27∼11.78; *p*=0.64) in African-Americans versus −6.63 mmHg (95% CI: −11.81∼−1.44; *p*=0.01) in non-African-Americans [15], indicating that the black race has a lower RDN response rate than the white. Although there were similar trends in 24-hour ambulatory SBP of these two subgroups, the difference did not reach the significance [24]. This might be partly explained by genetic factors, antihypertensive medications, especially vasodilators, and poor drug adherence in African-Americans [24, 25]. Notably, the change in office SBP in “nonresponder” African-Americans was −15.5 mmHg in the RDN group and −17.8 mmHg in the sham group (*p*=0.64), while the “responder” Caucasian manifested −15.2 mmHg in the RDN group and −8.6 mmHg in sham group (*p*=0.01) at 6 months [24]. Nevertheless, another post hoc analysis conducted by Flack et al. [25] concluded that African-American race itself was not an independent indicator associated with the decreases in (office) SBP. It was the improved medication adherence and/or medication regimen that decreased the office SBP in black race at 6-month. Hence, it should revaluate the difference in ethnics susceptible to renal denervation.

In addition, limited and small sample size as it was, the results of comparison between Asians and Americans from subgroup (Koreans) analysis of the Global SYMPLICITY Registry indicated that SBP reductions were greater in Koreans versus whites (−27.2 ± 18.1 mmHg versus −20.1 ± 23.9 mmHg; *p* = 0.002 (adjusted)) at the 12-month follow-up [26], implying a potential difference between Asians and Americans, which needs more data and trials to lift the veil. Noteworthily, the recent published REQUIRE trial [16], conducted in Japan and South Korea, reported a negative result between the RDN group by ultrasound and the sham group at 3 months (24-h ABPM: −6.6 mmHg for the RDN group versus −6.5 mmHg for the sham group). Satisfying though the BP reduction is (while less than RADIOSOUND-HTN [14] and RADIANCE SOLO [12] or TRIO [27] in the USA and Europe), such unexpected reduction in the sham group might be attributed to medical adherence or single blindness, which would be addressed in the future, as they reported.

### 2.3. Obesity

Obesity is characterized by the overactivated sympathetic nervous system, especially in the kidneys and skeletal muscular vessels [28]. Thus, obese patients with resistant hypertension are potentially ideal subjects for renal denervation treatment, which has been proposed in clinical trials [4, 12], especially in obese females [29, 30]. However, Id et al. found that the BP-lowering effect in patients with obesity was less pronounced after RDN [31] while HTN-3 reported no significant difference of BP reduction in patients with or without obesity [15]. To summarize, these correlations were performed between the patients' BMI and efficacy, while only BMI fail to distinguish muscle and fat, as well as fat tissue distribution. Hence, more detailed parameters of obesity are needed to recalculate the correlations between obesity and efficacy.

### 2.4. Age

**Figure 1:**
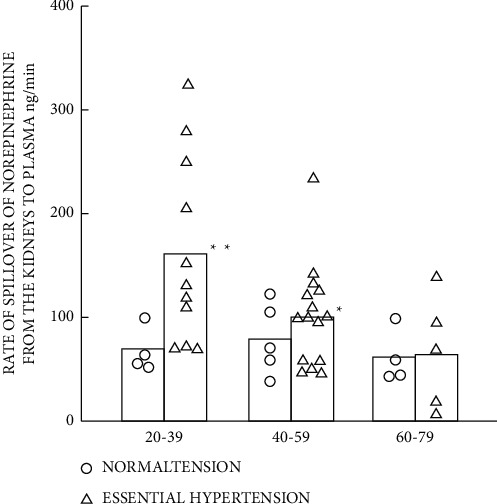
Influence of age (20–39, 40–59, and 60–79 years old) on renal norepinephrine spillover (ng/min) in normotensive and hypertensive patients. *Note.*^*∗*^*p* < 0.05; ^*∗∗*^*p* < 0.01. Reproduced from reference [32].

Age is also a nonnegligible factor in patient selection. Apart from the vascular alterations by aging (see Vascular Stiffness and Isolated Systolic Hypertension section), sympathetic nerve activity varies between the young and the old. Esler et al. [32] revealed the relationship between age and renal sympathetic nerve activity by measuring the rate of spillover of norepinephrine from the kidney in hypertensive patients and elucidating that renal norepinephrine spillover is greater increased in the young patients (aged from 20 to 39, Figure 1) compared with the middle age (aged from 40 to 59) and the old (aged from 60 to 79), while there was no significant difference in the sympathetic nerve activity across the age spectrum of the normotensive subjects. Such studies provided evidence for the young to undergoing RDN for more superior BP-lowering effect, which was consistent with some clinical trials [29, 33]. On the contrary, the difference did not reach the significance, while the trend was observed in HTN-3 (−5.73 mmHg for the young and 0.09 mmHg for the old, *p*=0.27) and in RADIANCE-HTN SOLO appendix.

### 2.5. Blood Pressure

Baseline characteristics of blood pressure were widely analyzed by correlations with the antihypertensive effect after RDN. As is shown in post hoc analyses, higher baselines of office SBP in HTN-3 [24] and the Greek Renal Denervation Registry [29], nighttime SBP in DENERHTN [34] and RADIANCE-HTN SOLO [35], as well as 24 h-DBP [36] predicted the BP-lowering effect after renal denervation. Notably, in the control group of DENERHTN, nighttime BP failed to predict the response to standardized antihypertensive drugs, eliminating the confounding factor of medication.

**Figure 2:**
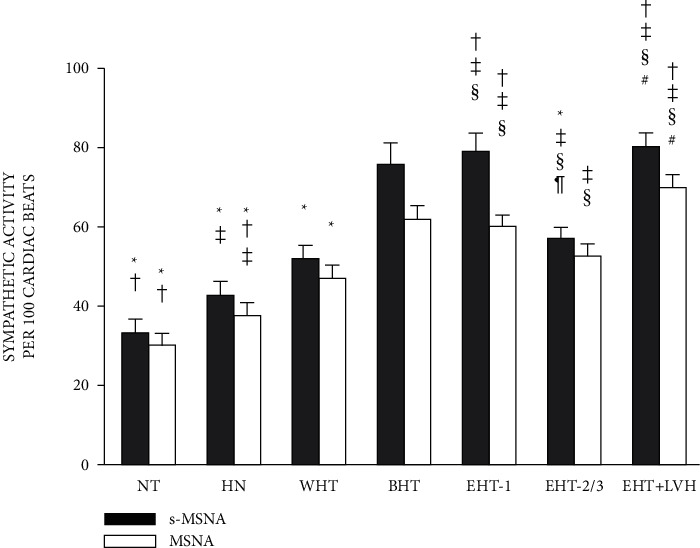
Multiunit muscle sympathetic nerve activity (MSNA) and single-unit muscle sympathetic nerve activity (s-MSNA), quantified per minute in seven groups. *Note.*^*∗*^*p* < 0.05 compared with borderline hypertension (BHT), ^†^*p* < 0.05 compared with white-coat hypertension (WHT), ^‡^*p* < 0.05 compared with normotension (NT), ^§^*p* < 0.05 compared with high-normal pressure (HN). ^¶^*p* < 0.05 compared with essential hypertension stage 1 (EHT-1), ^#^*p* < 0.05 compared with EHT stages 2 and 3; LVH, left ventricular hypertrophy. Reproduced from reference [37].

Also, Smith et al. [37] showed that, in essential hypertension, the magnitude of the overactivated sympathetic nervous system varies with its severity and complications and revealed that a greater increase in sympathetic activity occurred in borderline hypertension (intermittently over 140/90 mmHg), essential hypertension stage 1 (140–159/90–99 mmHg), relative to essential hypertension stage 2/3 (over 160/100 mmHg), compared with normotension and high normotension (Figure 2). Such results suggested potential candidates for renal sympathetic denervation. In other words, patients with early staged hypertension might be more suitable for RDN [37, 38]. However, the reason why the muscle sympathetic activity of patients with hypertension at stage 2/3 was less than that at the early stage may be the multidimensional regulation of blood pressure, e.g., baroreflex, which masked the real relationship between blood pressure and sympathetic activity.

### 2.6. Heart Rate

Though disputed, heart rate might be served as a sympathetic biomarker [39]. Michael Böhm et al. [40] revealed that patients from SPYRAL HTN-OFF MED with baseline 24-hour heart rate (HR) over the median 73.5 beat per minute (bpm) showed a significant reduction in average ambulatory SBP (−10.7 mmHg) at 3-month follow-up after RDN, comparing to those with below-median HR, whose BP-lowering effect was not significant. Similarly, in their another analysis [41], they reported office HR ≥ 70 bpm was associated with BP reduction (24 h-SBP: −6.2 mmHg, *p* < 0.001), but reduction of −0.1 mmHg for baseline office HR <70 bpm (*p* = 0.97). Notably, in SPYRAL HTN-OFF MED study, reduction of 24 h HR at 3 months reached the significant difference (−2.5 bpm), while it did not in the sham group (−0.2 bpm) [40]. Without confounder of *β*-receptor blockers in the OFF MED study, increased heart rate seems to be a reliable and readily attainable indicator for sympathetic hyperactivity. Similarly, Hoogerwaard et al. [42] proposed that decreased heart rate variability induced by renal nerve stimulation before and after RDN indicates a lower sympathetic activity after ablation, which was more pronounced in *β*-blocker naïve patients.

### 2.7. Antihypertensive Medication

Kandzari et al. [25] reported that the estimated change of SBP at 6 months was −6.39 mmHg (95% CI: −11.24∼−1.54, *p*=0.010) in HTN-3 pooled patients with administration of aldosterone antagonists versus +5.49 mmHg (95% CI: +1.26∼+9.72, *p*=0.011) in those with vasodilators, and both of them were more pronounced in the RDN group (−9.77 mmHg (95% CI: −15.83∼−3.72, *p*=0.002) of aldosterone antagonists versus +7.55 mmHg (95% CI: +2.38∼+12.72, *p*=0.005) of vasodilators).

### 2.8. Renin-Angiotensin System

The renin-angiotensin system (RAS) has been considered to be associated with sympathetic nerve activity and played a critical role in systematic blood pressure regulation [43]. In preclinical experiments and clinical trials, several studies have demonstrated the decreased RAS activity in the kidney (experimental, as well as upregulated ACE2/Ang (1–7)/Mas axis [44]) and plasma (experimental and clinical) after renal denervation [45–47]. Subsequently, Felix Mahfoud et al. [47] demonstrated higher baseline plasma renin activity (PRA ≥ 0.65 ng/mL/hour, compare with those PRA < 0.65 ng/mL/hour) was associated with a significantly greater reduction in office and 24 h-SBP at 3-month follow-up in patients with similar baseline blood pressure and absence of antihypertensive drugs.

### 2.9. Vascular Stiffness and Isolated Systolic Hypertension

Vascular function plays an important role in blood pressure regulation, and vascular stiffening is a major contributor to isolated systolic hypertension (ISH) and strongly associated with age [48], making it seemingly unsatisfactory to reduce blood pressure by denervation therapy. Although some trials excluded the patients with ISH for RDN studies, a study with large sample size of 1103 patients from SYMPLICITY HTN-3 and Global SYMPLICITY Registry showed a less pronounced office SBP reduction in the ISH group (−10.9 ± 21.7 mmHg) than the combined systodiastolic hypertension (CH) group (−18.7 ± 23.7 mmHg) at 6 months after RDN [49]. Notably, in patients with ISH, there was no significant difference observed between older (>65 years) and younger (<65 years) patients based on office SBP, indicating that the efficacy of RDN are more likely to associated with vascular stiffness rather than physiological changes by aging. This correlation has been further evidenced by Sata et al. [50]. They applied ambulatory arterial stiffness index (AASI, calculated as 1-regression slope of 24 h-DBP on 24 h-SBP [51]) to represent arterial stiffness and demonstrated that a lower AASI (<0.51) is an independent predictor of BP response to RDN, while patients with AASI over 0.51 showed no change in 24 h-SBP at 6-month follow-up. Besides, patients with the highest quartile AASI (>0.60) had a lower muscle sympathetic nerve activity than the other three quartiles. In summary, these studies imply that the BP-lowering effect of RDN in patients with ISH is more likely caused by neurogenic rather than biomechanical modulation.

Additionally, arterial stiffness could be represented by other parameters from RDN trials. Lurz and his colleagues [52] reported that invasive aortic pulse wave velocity, the gold standard for arterial stiffness [53], measured intraoperatively before ablation and was significantly higher in nonresponders (<5 mmHg in systolic daytime 24 h blood pressure) than that in responders (17.7 ± 4.7 m/s versus 14.4 ± 4.4/s, *p*=0.009). Furthermore, they reported noninvasive indicators of ascending aortic distensibility and (total) arterial compliance for measurements of stiffness by cardiac magnetic resonance to select potential responders [54]. Another study suggested that patients with lower central pulse pressure (below the median 55 mmHg) manifested a significantly greater BP reduction assessed by either office BP or 24 h-ABPM after RDN compared to those with higher central pulse pressure, indicating a lower degree of damage of the arterial vasculature [55]. Other biomarkers, such as galectin-3 [56], intercellular cell adhesion molecule-1 (ICAM-1), vascular cell adhesion molecule-1 (VCAM-1), and soluble receptor fms-like tyrosine kinase-1 (sFLT-1) [57], leading to stiffness or endothelial dysfunction, are also proposed to be predictive indicators of responders for RDN at baseline.

### 2.10. Baroreflex

Baroreflex is mainly involved in short-term regulation of blood pressure and associated with spontaneous fluctuations of arterial blood pressure and heart rate and baroreflex sensitivity (BRS) correlated closely with sympathetic activity [58, 59]. Zuern et al. [59] reported that attenuated cardiac baroreflex sensitivity, calculated by progressive elevation of SBP during ≥3 heart beats where R-R intervals simultaneously prolong, could identify those patients who are responders to RDN for treatment of resistant hypertension. Of note, although impaired BRS could distinguish the responders, it did not correlate with the magnitude of BP reduction after RDN.

### 2.11. Renal Artery Vasodilation

After complete renal denervation, reviewing postprocedural renal artery might predict the antihypertensive effect of RDN by sufficient destruction of renal sympathetic nerves which mediate vascular resistance, renin release, and sodium reabsorption [60]. Doltra et al. [61] and Chen et al. [62] demonstrated that renal artery vasodilation was observed after intervention in human and canine, and Chen further revealed that vasodilation was correlated with blood pressure reduction and plasma norepinephrine at 3-month follow-up. On the other hand, it is reported that transvascular pacing of aorticorenal ganglion in sheep produced ipsilateral renal arterial vasoconstriction without contralateral renal vasoconstriction [63], which might also provide a predictive indicator for successful RDN and procedural endpoint if vasoconstriction effect vanishes. They believed that aorticorenal ganglion pacing may elicit the hypertensive effect by afferents and the renal vasoconstrictive effect by efferent nerves. Combined these two experiments, renal vasodilation after RDN seems appropriate for prediction of successful RDN.

## 3. Procedural Factors

### 3.1. Ablation Sites

As is reported in previous studies, the number of ablation sites in each renal artery has been considered as a major influence factor for prediction of office SBP [24, 64], rather than 24 h-ABPM [13, 64, 65] after RDN. Furthermore, ablations at different segments of renal artery are also confounding factors affecting the outcome of RDN. Accumulating evidence suggests the more superiority of ablation at sites in the distal segment or branches (combined with main arteries) than that in proximal or main renal arteries alone by radiofrequency energy [66–68]. Inconsistent to these clinical studies, RADIOSOUND-HTN [14] failed to show the significance of radiofrequency denervation between main renal artery ablation and combined main artery and branch ablation. Another trial [69] indicated that denervation in proximal segments has similar efficacy and safety profile compared with full-length denervation.

Postmortem study suggested that despite fewer nerves surrounding the distal segment of renal arteries, the distance from nerves to the lumen was shorter than proximal segment [70], providing the rationale that in the same condition of ablation parameters, renal nerves traversing distal segments and branches would be destroyed more sufficiently. Hence, a higher energy is believed for a more complete denervation in proximal segments under the premise of safety because, theoretically, destruction of proximal nerves could consequently cut off the convergent pathway. Therefore, compared with limited penetration depth of 4–6.5 mm [71, 72], a deeper penetration of 7.5 mm [73] by ultrasound may explain the more superiority of ultrasound-based RDN in main arteries than radiofrequency in main stem from RADIOSOUND-HTN study. Despite this superiority, reduction of impedance during radiofrequency delivery could help the interventionist confirm the procedural success [72], which could partly address the insufficient ablation aroused by HTN-3.

However, García-Touchard et al. [74] revealed that in cadavers, more than half of the renal nerves reached the kidney by passing the main stem; in other words, these nerves ran tangentially and joined the distal segments or branches of renal arteries, called “late arriving nerves,” emphasizing the importance of ablation at distal arteries and branches. In conclusion, more rigorous-designed studies/experiments need to be performed to compare the strategies of device or energy-specific renal denervation on different segments of renal arteries.

### 3.2. Renal Nerve Stimulation

**Figure 3:**
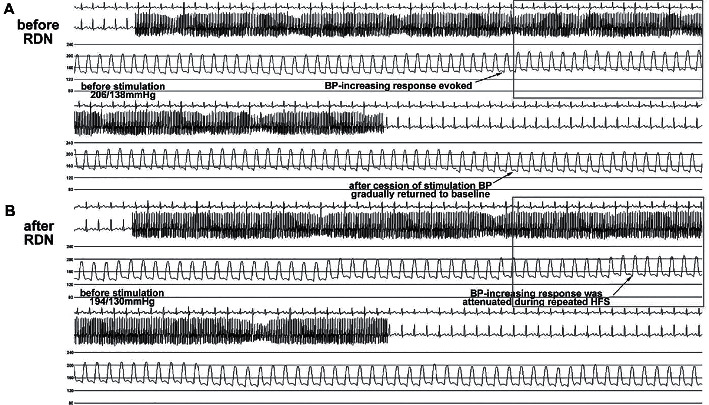
(a) An example of renal nerve stimulation evoking elevation of blood pressure before renal denervation; (b) the same stimulated site of (a) that elevation of blood pressure has been attenuated after renal denervation, implying a success in destruction of renal nerves. Reproduced from reference [76].

Renal nerve stimulation (RNS) has emerged as a feasible and promising method for mapping renal innervation to guide renal denervation since 2013, when Chinushi et al. [75] first introduced electrical stimulation into the renal artery for feasible exploration of renal autonomic nerve's functional location in canine. Subsequent animal studies [76, 77] also found elevated blood pressure response to RNS, while the increase in blood pressure response to RNS would be dramatically attenuated after ablation with one catheter for both stimulation and ablation, implying a success in destruction of renal nerves (Figure 3). Another study [78] proposed that the elevated blood pressure response during radiofrequency energy delivery in patients might be an intraprocedural predictor for the antihypertensive effect of RDN. The authors believed that renal sympathoexcitatory afferents were stimulated by radiofrequency energy, inducing an increase in blood pressure and implying successful location of renal nerves.

Consistent with animal experiments, preliminary clinical trials [79, 80] have evidenced the feasibility and safety in hypertensive patients on medication treatment, with far less number of ablation sites (4–6 sites per artery [79] versus 45.9 ± 13.7 sites per patients [10]) but more superior BP-lowering effects compared to SPYRAL HTN-ON MED (24 h-ABPM reduction from 153.3 ± 12.9/89.0 ± 3.5 to 135.0 ± 9.4/73.6 ± 13.5 mmHg [79] versus reduction of 9.0 ± 11/6 ± 7.4 mmHg with a mean baseline of 152.1 ± 7.0/97.2 ± 6.9 mmHg [10]). Furthermore, the amplitude of BP-elevation response to RNS [77, 81] and the magnitude of blunted response after RDN [77] were correlated with the antihypertensive effect postprocedure in both, suggesting that selective ablation at sites of greater elevated BP response could make RDN more predictable and efficacious. In summary, RNS could not only help to locate ideal ablation sites but also to assess whether renal nerves are completely denervated by repeated RNS at identical sites for prediction of the outcomes of RDN.

Although the detailed mechanism of about how electrical stimulation elicit blood pressure response is not fully understood, activation of renal afferents has been generally considered as the main contributor to immediate elevated BP response to RNS by projecting to the central nervous system down to peripheral sympathetic efferent nerves. The anatomy and physiological basis of renal innervation still remain limited and controversial. Sakakura et al. [70] revealed two nerve components in cadavers, efferent (stained by tyrosine hydroxylase, TH) and afferent (stained by calcitonin gene-related peptide, CGRP) fibers, and efferent nerve fibers were predominant (TH/CGRP ratio 25.1 ± 33.4). While van Amsterdam et al. [82] reported sympathetic nerves (73.5%, stained by TH), parasympathetic nerves (17.9%, stained by neuronal nitric oxide synthase, nNOS), and afferent nerves (8.7%, stained by CGRP) in cadavers, even though nNOS might not be the best candidate for labeling vagal nerves. Reasonably, Kiuchi et al. [83] categorized renal nerves as “pressor” or “depressor” based on the nerve functions regulating blood pressure in response to RNS, in accordance with the present studies that, apart from elevation response, reduced blood pressure during RNS was also observed in experimental and clinical studies [84, 85].

Similar to the “late arrival nerves” discussed above, even if there might not be parasympathetic innervation of the kidney, they may bypass renal artery and be activated by electrical stimulation, or denervated by ablation energy. Excessive ablation at sites of reduced blood pressure response might be one of the factors leading to an increase in blood pressure at follow-up postprocedurally, which needs well-designed animal experiments to validate. Therefore, renal nerve stimulation becomes a vital auxiliary technique to locate sympathetic-excitatory sites to ablate and sympathetic-inhibitory sites to avoid.

Not surprisingly, there are also some limitations of RNS-guided RDN. Firstly, RNS may cause pain to patients under conscious state to maintain afferent and autonomic function. Secondly, a sufficient duration of RNS for targeted ablation sites greatly prolongs the operation time compared to the conventional RDN procedure. Thirdly, plausible blood pressure response needs to be preciously identified by experienced operators, especially in patients who manifest few responsive sites and are not appropriate for RDN.

### 3.3. Denervation Devices

**Figure 4:**
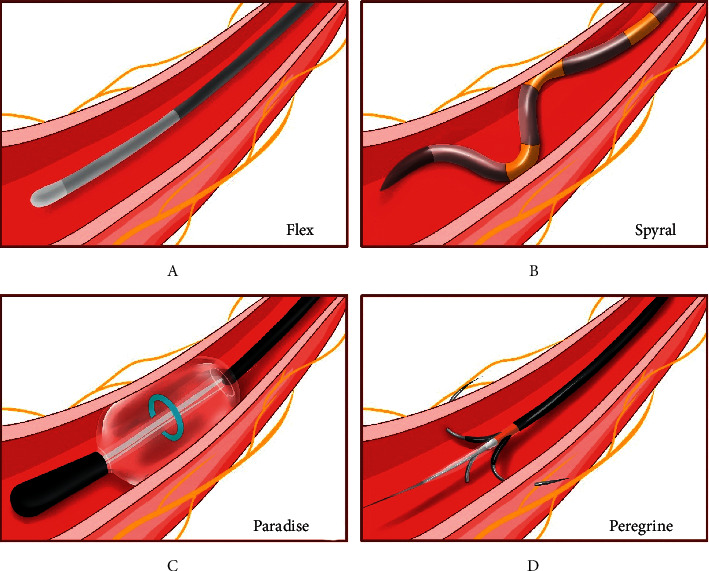
Different catheter-based renal denervation devices for reduction of sympathetic nerve activity and blood pressure.

Symplicity Flex catheter (Figure 4(a)), the first generation RDN system, is a monoelectrode radiofrequency device used in SYMPLICITY HTN-1–3 and DENERHTN [4, 5, 13, 15], with inconsistent conclusion about the efficacy of RDN, even though suboptimal patient selection, medication adherence, and technical failure were blamed for its unexpected neutral results in HTN-3 [86]. To address the shortcoming in HTN-3, the second generation device Symplicity Spyral catheter system emerged with 4 gold electrodes in four quadrants for circumferential ablation (Figure 4(b)). The SPYRAL HTN-ON MED [10] and OFF MED pivotal [11] were designed to compare patients with or without medication in the RDN group and the sham group, and patients with antihypertensive drugs manifested more reduction in 24 h-ABPM than OFF MED study, indicating that patients would benefit more when medications and renal denervation are combined.

The Paradise system (Figure 4(c)) utilizes ultrasound energy emitted to the arterial wall circumferentially by a cylinder-shaped energy-emission probe housed in an inflatable balloon without direct contact with the endarterium. 2–4 sites recommended in each artery and 7-second energy delivery in each site shorten the procedure time. As is shown in RADIOSOUND-HTN [14], reduction in daytime SBP was greater in ultrasound ablation of the main artery than radiofrequency ablation of the main artery, revealing the superiority of ultrasound-based RDN. Interestingly, similar magnitude of reduction in ambulatory SBP was observed in patients undergoing RDN in the “on med” group (−8.0 mmHg in RADIANCE-HTN TRIO) [27] and “off med” group (−8.5 mmHg in RADIANCE-HTN SOLO) [12], while patients in the sham group with standardized triple fixed dose combination pills (TRIO) showed a more decreased daytime SBP than patients without medication (SOLO), implying that renal denervation by ultrasound could lead to an analogous antihypertensive effect regardless of medications or not.

The Peregrine catheter (Figure 4(d)) is designed for chemical ablation by injection of absolute ethanol at a low dose, a potent neuritis and neurolysis agent [87], to adventitia and periadventitial tissue for renal denervation [17, 87]. Peregrine contains three microneedles (220 *μ*m) which are placed in the body of the catheter and could be deployed into renal arteries by the control handle for alcohol delivery. Both the preclinical experiment [87] and clinical trials [17, 88] have evidenced the efficacy in a dose-dependent manner with safety profile. Moreover, another multicenter, sham-controlled, randomized trial in the absence (TARGET BP OFF MED, NCT03503773) and presence (TARGET BP I, NCT02910414) is ongoing, using the Peregrine system kit, which would extend the scale of sample size and further validate its safety and efficacy in a more rigorous-designed way.

**Figure 5:**
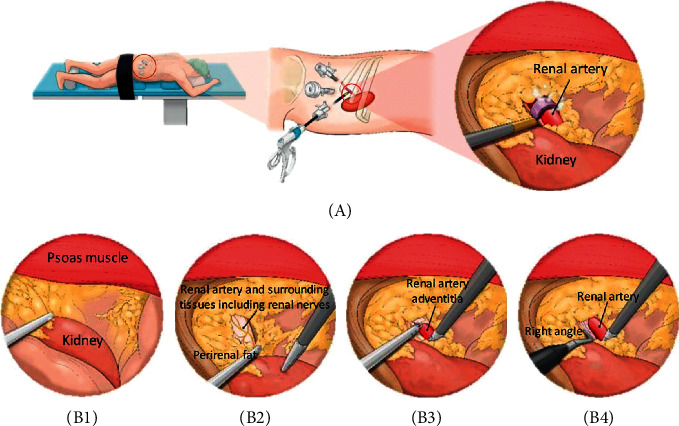
Flowchart of laparoscopic renal denervation. (a) Modified prone position for laparoscopic RDN. (b) Schematic to show the steps of laparoscopic RDN; (B1) dissection between psoas muscle and Gerota's fascia; (B2) removal of fat tissues around the renal hilum and identification of connective tissues surrounding the renal artery including renal sympathetic nerves; (B3) removal of periarterial connective tissue using a laparoscopic monopolar hook electrode; (B4) securing space to insert the laparoscopic ablation instrument using right angle dissector. Reproduced from reference [3].

The laparoscopic renal denervation system (Figure 5) [3, 89] combined laparoscopy and radiofrequency catheter which was characterized by flexible electrodes at the tip head and controlled by the actuator to curve and wrap the adventitia of renal arteries for 360-degree direct ablation (instead of energy transmission from intima). Although the proof-of-concept study in the porcine model showed the validity of laparoscopic RDN in main, branch and accessory arteries via the retroperitoneal route, the sample size is too small, and they did not report device-related surgical events. Hence, more preclinical studies are needed to probe to the effects and safety of laparoscopic-based RDN and provide strong rationale for clinical trials.

The ConfidenHT system (Pythagoras Medical Ltd, Israel), using RNS for mapping renal nerves, contains a console and a dedicated catheter which consists of a flexible multielectrode in a basket shape. Notably, the preprogrammed stimulation is performed at 2 mA, followed by 4 mA for 2 min without causing discomfort to the patients under conscious condition. However, only a few participants were enrolled for assessment of feasibility without ablation [90]. On the other hand, a multicenter RCT of RNS-guided renal denervation using the SyMapCath I catheter and SYMPIONEER S1 Stimulator/Generator (SyMap Medical Ltd., China) is expected to be presented in late 2022, which has enrolled all the patients with medication to investigate the effects of RNS-guided renal denervation (SMART Trial, NCT02761811).

## 4. Discussion

Renal denervation has been validated in a number of clinical trials for its safety and efficacy and not only confined to treatment of resistant hypertension. However, such benefits are not shared by all subjects, some of which even manifest more severe hypertension after RDN. Though many indicators have been listed, the leading clinical need to be met is the patient selection, which is critical for clinicians to bring benefit for ideal candidates, especially in younger and early staged primary hypertensive patients with better vascular function, especially those with obesity, higher baseline blood pressure and/or heart rate, and avoid to burden nonresponders. Moreover, patient preference seems to be an instant and readily information for patient selection and even may influence the outcomes of the efficacy of renal denervation. From the survey of the patient preference for renal denervation conducted in Europe and Asia [91–93], the patients characterized by younger, males, higher blood pressure, presence of heart failure, or other side effects of medications, and, most importantly, poor antihypertensive drug adherence are more likely to choose device therapy for management of hypertension.

Correlations between baseline blood pressure and efficacy after RDN are complicated and various, while blood pressure responses to renal nerve stimulation and radiofrequency energy seem promising, and another shot of RNS after renal denervation at the same site could confirm a successful ablation if the blood pressure remains relatively stable. Last but not the least, improvement of devices and strategies is expected to be more direct and sufficient to overcome such limitations.

Patients would benefit more from RDN when complicated with chronic obstructive pulmonary disease [94], chronic kidney disease [94, 95], and especially obstructive sleep apnea-hypopnea syndrome [96]. Obstructive sleep apnea is highly correlated with increased sympathetic tone and renin-angiotensin system mainly by hypoxia/hypercapnia [97]. Continuous positive airway pressure is the most common therapy for these patients, but has little effect on controlling blood pressure and cardiovascular event. Hence, patients suffering from both hypertension and obstructive sleep apnea become more suited for renal denervation.

Besides the predictors mentioned above, other studies have also proposed some fragmented markers for responders: patients with lower absolute values of activated double negative T cells and lower but more stable values of total CD8+, CD4+, and naïve T CD8+ cells [98], increased serum vitamin D concentrations [99], reduced serum brain-derived neurotrophic factor levels [100], serum IL-6 levels [101], decreased plasma midregional proadrenomedullin [102], and intraprocedural reduced venoarterial norepinephrine gradient [103].

Many as the indicators are, there is lack of an actual gold standard or prediction model for inclusion of responders, the threshold of which remains to be addressed by a larger sample size and racial difference. Renal nerve stimulation seems much promising, while RNS needs to be further verified by large randomized controlled clinical trials. Also, a dedicated algorithm could be built in the ablation system to help interventionalists for decisions on ablation or avoidance. Nevertheless, above predictors should be further evidenced, and new indicators or treatment devices might be necessary.
